# Recommendations for a Pediatric Pain Education Curriculum for Physical and Occupational Therapists: Scoping Review and Survey

**DOI:** 10.3390/children8050390

**Published:** 2021-05-13

**Authors:** Marjan Laekeman, Axel Schäfer, Martina Egan Moog, Katrin Kuss

**Affiliations:** 1 Physiological Psychology, Otto-Friedrich-University of Bamberg, 96045 Bamberg, Germany; 2 Faculty of Social Work and Health, University of Applied Science and Arts, 31134 Hildesheim, Germany; axel.schaefer@hawk.de; 3 Precision Ascend Pain Management, Melbourne, VIC 3101, Australia; martina.egan.moog@gmail.com; 4 Department of General Practice/Family Medicine, Philipps-University Marburg, 35043 Marburg, Germany

**Keywords:** pediatrics, pain, curriculum, education, occupational therapists, physical therapists, review, survey

## Abstract

Specialization training for physiotherapists, occupational therapists, and sports therapists involved in pediatric pain is scarce and curricula are rarely published. The objectives of this study are twofold: firstly, to perform a scoping review to derive important contents for a pediatric pain education curriculum for specialized pain therapists. Secondly, to conduct a survey on specific contents in curricula currently used by pain experts and to obtain their evaluation regarding the importance of such contents for a specialized curriculum. The review substantiated the importance of a specific curriculum in pediatric pain education, but provided little information on adequate contents. In the survey, 45 experts in pediatric pain education confirmed that specific curricula and specialized contents for pediatric pain education are missing. Their answers give a well-defined picture of the specifics needed in the interaction with a pediatric population. The most important items they classified were e.g., the biopsychosocial framework and the impact of pediatric pain on daily life. Those expert ratings were in line with the recommendations of pediatric pain management guidelines. Further curriculum work in an interdisciplinary, international network is highly recommended.

## 1. Introduction

All children in different age groups experience pain at some stage [1]. Infants are often exposed to procedural pain [2], and older children or adolescents often suffer some form of chronic pain. A systematic review reported prevalence rates for headache between 8–83%, abdominal pain between 4–53%, back pain between 14–24%, and musculoskeletal pain between 4–40% [3]. Without adequate pain prevention or pain treatment, early pain experiences can have livelong consequences, with impact on quality of life, specifically interfering with school and sport performances, appetite, mood or sleep [4] and can lead to increased pain sensitivity later in life [5].

Current evidence that physical activity/exercise training may be effective for reducing or preventing pain is often based on data extrapolated from studies on adult populations [6,7]. Although there is still need for more high-quality studies in pediatric populations [1,8], there is growing evidence suggesting that exercise/physical activity interventions are also effective for reducing pain and improve physical functioning in this vulnerable group [9,10].

In the rehabilitation of pediatric chronic pain, exercise and physical activity promotion are key elements, hence physiotherapists (PTs) and/or occupational therapist (OTs) form an integral part of the management team in existing pain programs [11,12,13]. There is increased support to involve PTs beneath pediatricians and clinical child psychologists regarding coordinated interventions in intensive interdisciplinary pain treatment programs [13]. A review focusing on specialized pediatric rehabilitation programs with severe disabling chronic pain depicted PT interventions in all of the analyzed programs and OT Interventions in seven of the nine analyzed programs [12].

However, the quantity and quality of the therapists’ training are not standardized with regard to pediatric pain management, and it is unknown how much of the required knowledge and skills are already integrated in existing basic education programs. Unfortunately, basic pain education for health care professionals who regularly work with children is still inadequate despite a large body of literature [14], and deficits regarding basic pain topics in physiotherapy and occupational therapy curricula have been noted [15,16,17]. The phenomenon of insufficient practice transfer is a well-known challenge that also needs to be addressed at an early stage of training [14].

Yet an overview of quantity and contents of pediatric pain education integrated in basic educational curricula for physiotherapists (PTs), occupational therapists (OTs), and sports therapists (STs) still needs to be established.

The general objectives of this study are twofold: firstly, to perform a scoping review to derive important contents for a pediatric pain education curriculum for specialized pain movement therapists. Secondly, to survey the presence of such specific contents in the curricula currently used by pain experts and to get their evaluation regarding the importance of such contents for a specialized curriculum.

## 2. Materials and Methods

### 2.1. Theoretical Framework of the Study

Due to the scarcity of specialized pediatric pain management training among therapists, curricular adjustments in educational programs are recommended [1,14]. Differentiated knowledge and understanding of pediatric pain are essential in order to make the correct decision regarding a child centred pain management.

**Figure 1:**
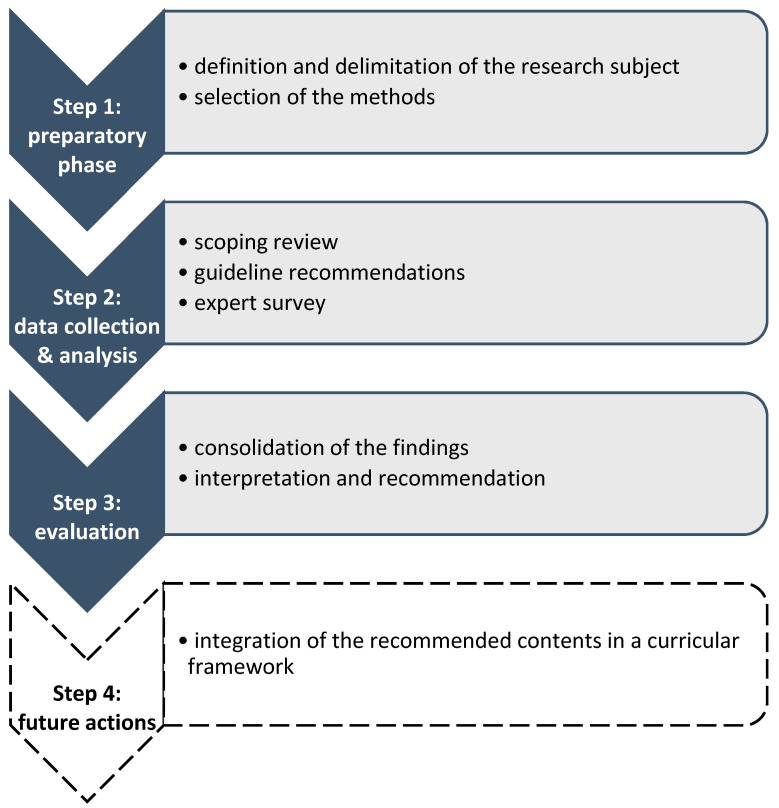
Theoretical framework of the study. The grey parts of this figure have been finished and are reported in this article. The white part remains to be done in the future.

Curricula should implement contents with high relevance regarding this educational topic. Therefore, a decision was made to focus this study on the derivation of appropriate topics for a pediatric pain curriculum using a scoping review, guideline recommendations and an expert survey. This developmental study was performed, following the principles of the educational design research [18]. The different steps of the study are depicted in Figure 1 and described in depth in this section. Step 4 presents possible actions for the future.

### 2.2. Scoping Review

This scoping review was undertaken to get an overview how pediatric pain is addressed in PT/OT/ST curricula/education and to derive essential contents for a specialized pediatric pain curriculum for therapists involved in pediatric pain. The review was carried out based on the recommendations of “PRISMA Extension for Scoping Reviews“ [19,20].

#### 2.2.1. Search Strategy

From 6–10 December 2020, three independent authors (ML, KK and AS) performed the search in five databases (Medline [PubMed], Cochrane, CINAHL, PEDro, OTSeeker). Different combinations of the following terms were used: pain; curriculum; physiotherapy; occupational therapy; sports therapy; exercise therapy; sports medicine. The literature search strategy of Medline (via Pubmed) is exemplarily provided in Box 1:

Box 1 **Literature search strategy Medline via Pubmed (search 6/12/2020).**

   (“Pain”[Mesh] OR “pain“[tiab]) AND (“Curriculum”[Mesh] OR “curricul*”[tiab]) AND ((((((((“Physical Therapy Specialty”[Mesh]) OR “Occupational Therapy”[Mesh]) OR “Exercise”[Mesh]) OR “Exercise Therapy”[Mesh])) OR “Sports”[Mesh]) OR “Sports Medicine”[Mesh]) OR (“Physiotherap*” OR “Physical therap*” OR “Occupational therap*” OR “sports therap*”))

The authors assumed that there are hardly any specific curricula for pediatric pain management, therefore a broader search strategy was chosen. Wildcards and Boolean operators were used and all types of studies were included, without any date restriction. The search strategy for PubMed as depicted above was adapted for the other databases. Also, reference lists of the selected articles were reviewed to identify further important papers (backward citation tracking).

#### 2.2.2. Selection Criteria

After de-duplication, the abstracts and eligible full texts were assessed by two authors (ML, KK) independently according to predefined criteria. Inclusion: articles written in English, German, French or Dutch; studies that refer to pain education curricula for movement therapists (physiotherapists, occupational therapists, sports therapists); studies reporting curriculum and/or education training contents regarding pain management for those professions; mention of pediatric/adolescent/child pain, or pain in vulnerable people, or pain across the lifespan. The exclusion criteria represented editorials or congress abstracts, or when no full text was available.

#### 2.2.3. Data Extraction and Critical Appraisal

**Figure 2:**
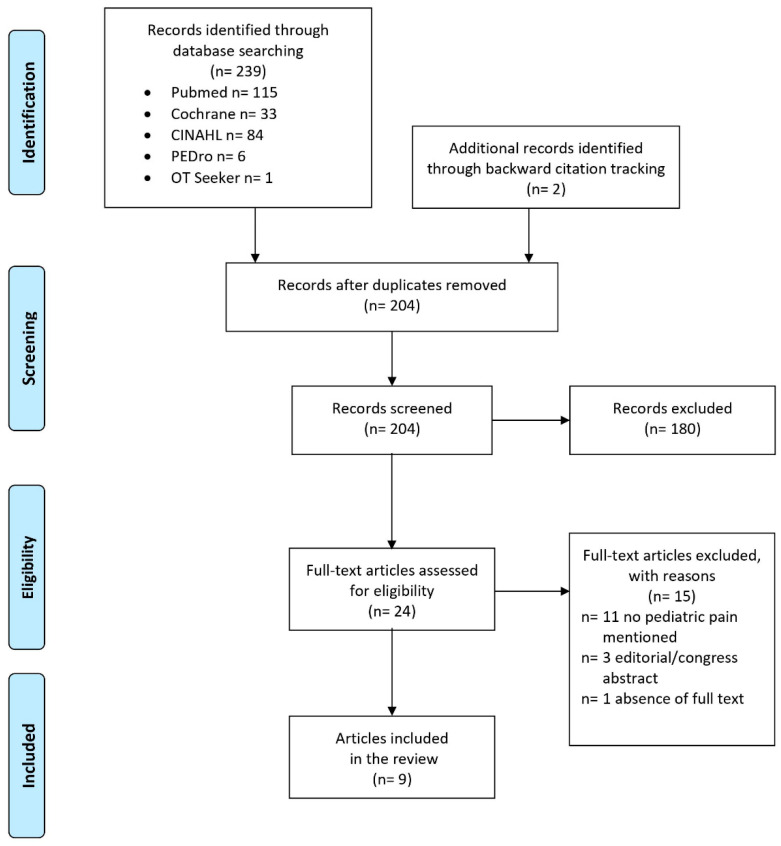
Flowchart of the study selection process.

Data were extracted from the texts, tables, figures and supplement documents of the selected publications. Two investigators independently reviewed the articles and disagreements were discussed until a consensus was reached (ML, KK). The flowchart for the selection and screening method is provided in Figure 2.

Data charting was done in a calibrated form that was developed and tested by two authors (ML, KK) before their use. Important contents of the included studies were selected and entered into this predefined table.

Since existing questionnaires for the critical appraisal of surveys did not exactly reflect criteria suitable for the evaluation, a special checklist for the quality grading was developed.

### 2.3. Survey

Since the literature review did not provide enough information of meaningful curricular contents, a survey among pediatric pain experts and/or pain educators was conducted using a self-constructed questionnaire for an online-survey.

#### 2.3.1. Development of the Survey Instrument

In line with the analyzed studies in our review, our questionnaire was constructed based on the general pain curricula developed by experts from the International Association for the Study of Pain (IASP), the Interprofessional [21], the Physical Therapy [22], and the Occupational Therapy Pain Curriculum [23], and also the European Pain Federation (EFIC) Core PT Curriculum [24]. However as these curricula are not focused specifically on pediatric pain education, proposed contents for a specific PT/OT curriculum for pediatric pain education were mainly derived from guidelines and two best evidence reports regarding pediatric pain, which included at least parts for physical and/or occupational therapy (*n* = 7) [11,25,26,27,28,29,30]. The largest part of the derived items is depicted later in the results section.

The online questionnaire included a cover sheet with information regarding the background and the aim of the survey, and guarantee of anonymity and data protection. The first part of the survey included six questions to collect information about characteristics of the responders (country, profession, educational level/qualification, work setting, involvement in curriculum development). Further eight questions asked for information about possibly existing PT/OT courses in pediatric and/or general pain management in the national IASP chapters. Two free text questions asked which learning objectives and main core competencies should be acquired by PTs and OTs in a specialized pediatric pain course.

The second part of the questionnaire was structured analogous to the IASP Curricula in four areas: (1) Multidimensional nature of pediatric pain (13 items); (2) Assessment and measurement of pediatric pain (26 items); (3) Management of pediatric pain (27 items); (4) Specific clinical conditions in pediatric pain populations (two items). The proposed contents regarding the examples of specific pediatric assessment tools and special treatment considerations were compiled from two authors (ML, KK) in consultation and appraisal with the two others authors (AS, ME). Regarding each proposed content for the four curricular areas, we asked if it was covered in the responder’s possibly used curricula (is covered in our curriculum; is not covered in our curriculum; no specific curriculum available). Further it was investigated how important they considered each content (this item is very important; this item is important; this item is less important; this item is not important at all).

In an extra box the participants were invited to list any additional contents for each area they considered important. Another extra box was added for possible further comments. The online survey was constructed and conducted by means of the software SoSci Survey (version 3.2.23) [31]. The survey was pre-tested for comprehensibility and adequacy in a group of pain experts (*n* = 10), who suggested only some minor changes. The final survey was sent on 9 February 2021 to the IASP Organization with the request for assistance in dissemination.

#### 2.3.2. Participating Experts

Addressees for our survey were defined as individuals being considered as experts in pediatric pain treatment and/or in pain education. With support from the IASP, the survey was at first distributed among the members of the Special Interest Groups ‘Pain in Childhood’, ‘Pain, Mind and Movement’ as well as ‘Pain Education’. All initially invited persons were asked to share the invitation and the weblink to other experts of their network. The survey was conducted in the period of 12 February till 12 March 2021.

#### 2.3.3. Analysis

For quantitative data, descriptive statistics (frequencies and percentages) for each category were calculated. All statistical analyses were performed with SPSS software (v 25.0; IBM Corporation, Armonk, NY, USA). A summarizing content analysis according to Mayring [32] was chosen to analyze ‘free text responses’ for participants’ personal opinions about ‘learning objectives’ and ‘main core competencies’ as well as for additional suggestions for the 4 areas regarding curriculum contents. The responses were paraphrased, generalized, and a first coding scheme was built deductively (the defined 4 areas) and augmented by inductively emerging new aspects; quotes are presented as examples. The coding and analysis were led by KK, and checked for validity and reliability by ML. Discussion of findings and interpretations within the research team helped to ensure credibility and trustworthiness. The analysis was assisted by MAXQDA software for qualitative data analysis, version 12 (MAXQDA. © 1989–2016, VERBI Software Consult Sozialforschung GmbH, Berlin, Germany).

## 3. Results

### 3.1. Results of the Scoping Review

#### 3.1.1. Study Selection

The literature search delivered 239 records; two additional records were identified through backward citation tracking (Figure 2). After removing duplicates, 204 records were screened from which 180 were excluded because they did not meet the inclusion criteria. After assessing the full texts of the remaining 24 articles, another 15 articles were excluded with reasons (*n* = 11 no pediatric pain mentioned, *n* = 3 editorial or congress abstract, *n* = 1 absence of full text). Finally, nine articles published between 2001 and 2020, fulfilled the inclusion criteria and were taken into account in the analysis [33,34,35,36,37,38,39,40,41]. An update of the literature search on 27 April 2021 in Medline, Cochrane, CINAHL, PEDro and OTSeeker databases delivered no new relevant records.

#### 3.1.2. Study Characteristics and Contents

The included articles (*n* = 9) contained general information about approximately 278 educational pain programs in six different regions. The results of the included studies are summarized in Table 1.

Most represented were pre-licensure/undergraduate programs from the USA [33,34], USA and Canada [35], North America and Canada [36], Canada [37,38], Australia [39], Spain [40], the Netherlands and international experts [41].

Most of the studies focused on PT programs [33,35,36,38,39], one on an OT program [34]. The remaining three programmes included several disciplines, among others also OT and PT [37,40,41]. Unfortunately, the data base search did not reveal any studies with information about a pain curriculum in sports therapy programs, thus we excluded this group from further investigations.

Different aspects of curricula were analyzed in the studies. Most of them carried out a survey to derive information about the contents and/or extent of pain education in current educational programs [33,34,36,37,38,40].

All the studies based their evaluation on one or more IASP Curricula [21,22,23]: either the interprofessional curriculum [40,41], or the PT Curriculum [33,38,39], or PT Curriculum and OT Curriculum [36], or PT and interprofessional curriculum [35], or all three [34,37]. Nevertheless, the IASP curricula were not universally followed in the educational programs: some of them implemented only a part of the items from the IASP domains cf. [33,36,38,40,41]. Most of the pain contents were embedded in the educational therapists’ programs and a few studies depicted some stand-alone pain courses (e.g., [33,36,37,38]). The total hours of pain contents varied greatly between the programmes, with a range of 4–129 h. Different groups were targeted in the evaluations: faculty directors or faculty members, pain experts, pain educators or students.

#### 3.1.3. Pediatric Pain Contents

Authors criticized that pediatric pain items were given too little consideration in the existing courses, and programs including specific pediatric pain contents were scarce [36,37,38,40]. For example, one study stated that 28.6% of the PT programs addressed assessment and management of pediatric pain in sufficient depth [38].

#### 3.1.4. Critical Appraisal of the Included Studies

Existing questionnaires for the critical appraisal of surveys do not reflect criteria suitable for our evaluation. Therefore, 10 questions from existing questionnaires and reporting guidelines were derived for the evaluation [42,43,44]. Table 2 shows that all the studies almost meet all of the appraisal criteria with the exception of 2 publications that did not exactly apply a survey methodology [35,39].

### 3.2. Results of the Survey

#### 3.2.1. Sample Characteristics

A total of *n* = 45 international pain experts from 18 different countries responded to the survey: European countries (*n* = 18; 40%); Australia (9; 20%); North-America (8; 17.8%); Africa (4; 8.9%); South-America (2; 4.4%); Asia (1; 2.2%); unknown (3; 6.7%). Concerning the professions, the most highly represented were physiotherapy (*n* = 28; 62.2%) followed by medicine (6; 13.3%), occupational therapy (4; 8.9%), psychology (3; 6.7%), nursing experts (2; 4.4%), and one response from clinical pharmacology (1; 2.2%) and from epidemiology (1; 2.2%), respectively. Regarding academic qualifications most of the respondents had a PhD/doctoral degree (*n* = 20; 44.5%), followed by a Master’s degree (11; 24.4%), Bachelor’s degree (5; 11.1%), or other (e.g., vocational school level, 5; 11.1%), 4 (8.9%) had a doctoral degree and another 4 were habilitated (8.9%). A high proportion of responders (73.3%) worked in a pain management setting, and 80% worked with children suffering from pain, with more than half of experts working with chronic pain and a negligible minority with acute pain. The responders were to a great extent (*n* = 29; 64,4%) involved in delivering (developing or teaching) a pain curriculum for health care professionals.

#### 3.2.2. Specialized Pain Management Courses

Only 4 responders (*n* = 8.9%) confirmed positively that their country chapter provides stand-alone specialization courses for pediatric pain management, 1 for PTs, 1 for OTs, and 2 for both, with a range of 2–17 h. Regarding generalized specialization courses for pain management, 16 (35.6%) of the participants confirmed positively that their country chapter provides such specialization courses, among 6 of such a course comprised >80 h. Overall, a range from 1–100 h was stated. Among 7 of those general courses stated above include a specific pediatric pain module with a range from 1–12 h.

To the open questions regarding main learning objectives and main core competencies for a specialized pediatric pain educational course, divergent answers were supplied, most of them coincided with the items already listed in our proposed contents for the curriculum.

Regarding learning objectives, the biopsychosocial approach appeared in almost all the answers and foundational knowledge of (pediatric) pain neuroscience/neurophysiology, in particular the differences between children’s and adult’s pain presentation, were mentioned. Further several answers refer to the particularly attentive way of dealing with this vulnerable group of pain patients and the importance of involving the environment (parents, school, peers) in the pain management. Some examples:

*“Understanding the neuroscience behind chronic pain and learning different ways to speak of it to families and children using metaphors, stories, toys, etc. …”*

(#150; OT)

*“… Difference in pain management in children vs adults”*

(#180; Medicine)

*“… it is important to take into account how the child experiences pain, and whenever possible to ask them and let them “tell” what their day-to-day life is like, of course, without losing sight of the family’s opinion…”*

(#193; PT)

*“…Considerations of language and it’s use, ways to simplify and enhance interventions to be understood by young people… The role of parents, attachment and family system functioning on pediatric pain…”*

(#352; OT)

Regarding the core competencies, it was also noticeable that emphasis was placed on competencies related to communication and educational skills in handling with these vulnerable patients and their families, e.g.,:

*“… empathy and feeling with the problems of acute and chronic pain in children …”*

(#248; Nursing)

*“… Be able … to coach the child and her/his family in adopting valued life goals.”*

(#267; Psychology)

*“… Affinity with this population, determination, enthusiasm and not careful in treating pediatric pain…”*

(#286; PT)

Furthermore, importance of interdisciplinary work and training was clearly advocated, e.g.,:

*“It would be nice if there was specific training, not just for physical and occupational therapists, but for a whole multidisciplinary team...”*

(#248; Nursing)

*“Ability to work in concert with a multi-disciplinary team including understanding how to communicate and maximize collaboration with physicians and psychologists.”*

(#133; PT)

These comments were also reflected in the answers to the closed questions.

**Figure 3:**
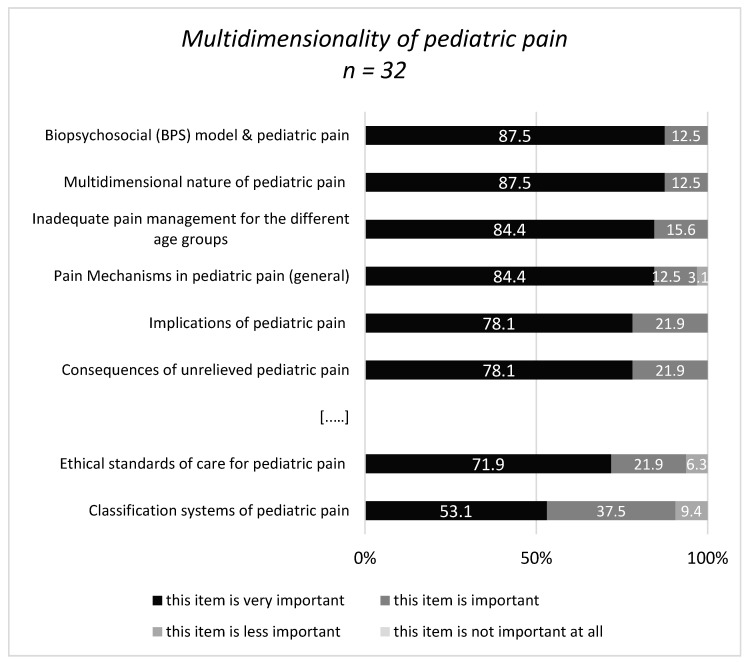
Response categories with highest/lowest accordance in area 1 (Multidimensionality). […..]: omission of items in the medium range.

**Figure 4:**
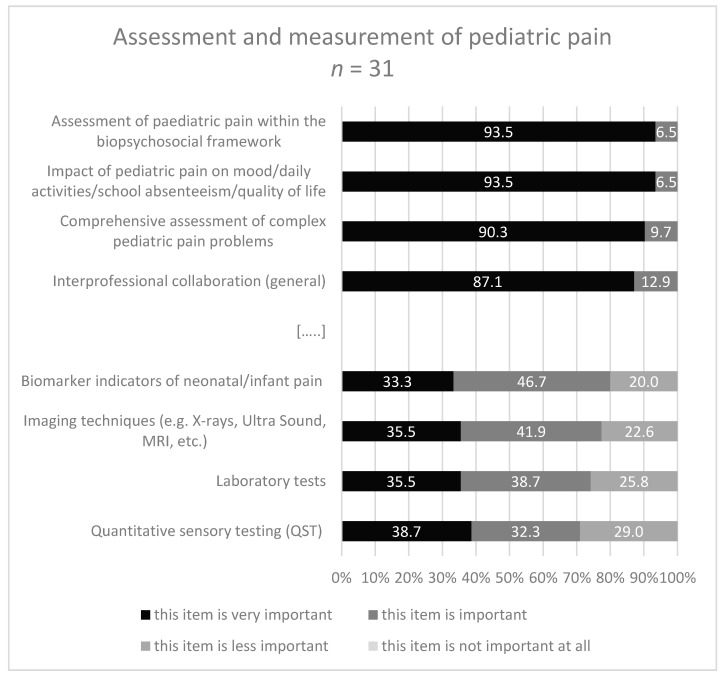
Response categories with highest/lowest accordance in area 2 (Assessment). […..]: omission of items in the medium range.

**Figure 5:**
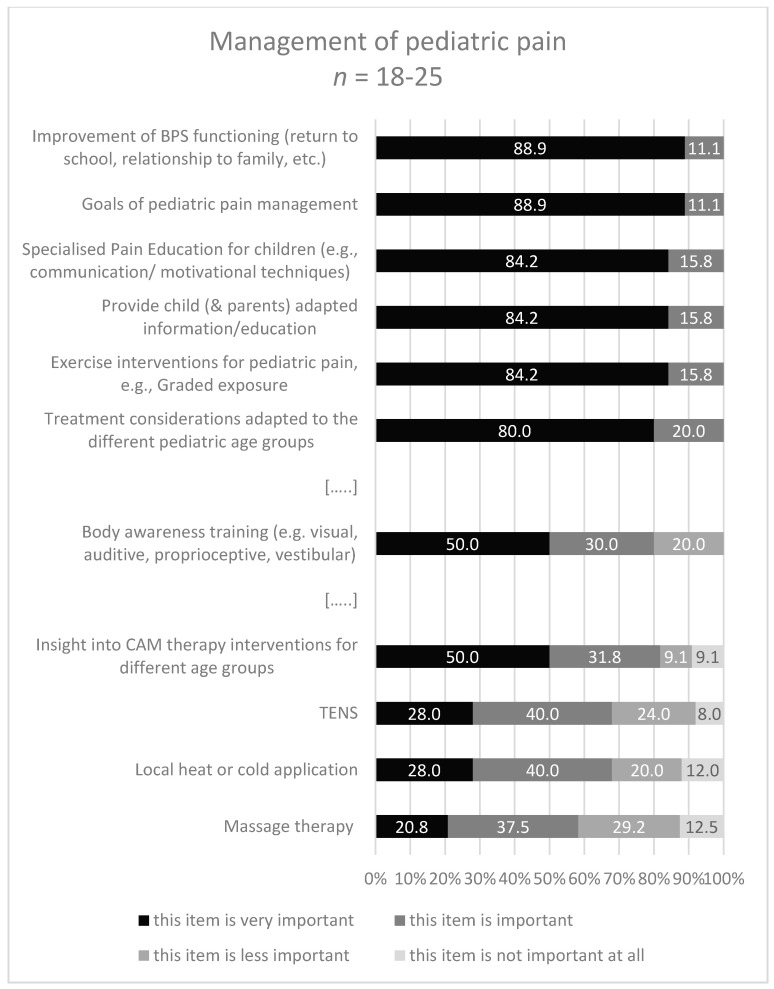
Response categories with highest/lowest accordance in area 3 (Management). […..]: omission of items. BPS: biopsychosocial; CAM: complement & alternative therapy interventions.

**Figure 6:**
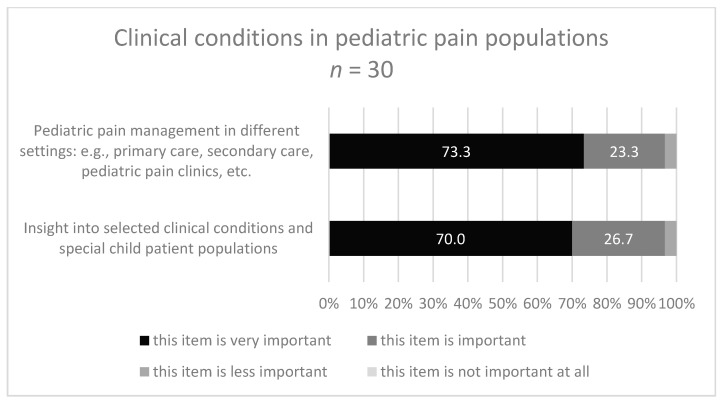
Response categories with highest/lowest accordance in area 4 (Clinical conditions).

To the question: “Which contents … are covered in your curriculum and/or do you rate as important for a specialization curriculum for pediatric pain management specific for PTs and OTs.?” 18-32/45 experts gave a response (Figure 3, Figure 4, Figure 5 and Figure 6). About half of them stated that they have no specific curriculum and only a part of the responders stated that they covered partially some items in their curriculum.

Regarding importance of the contents most of the responders classify the proposed contents in the categories ‘important’ or ‘very important’.

Within the first area ‘Multidimensionality of pediatric pain’ the content definition of pain occurred most frequently (50%) and the content ‘inadequate pediatric pain management for the different age groups’ was the most frequently not covered content (35.7%).

The contents with the highest percentage in the category ‘very important’ were ‘the biopsychosocial model and the multidimensional nature of pediatric pain’ (both 87.5%). Further ratings within the multiple, divergent response categories are illustrated in Figure 2 with regard to the most and least important items, respectively.

The experts were also invited to add suggestions for further contents for this area.

Once again, the biopsychosocial framework was in the foreground and the consideration of childhood development trajectories was mentioned, e.g.,:

*“… Attachment and role of developmental trauma on development (including how this impacts threat detection) …”*

(#352; OT)

*“… vulnerable populations (indigenous, remote and rural regions, co-morbid mental health, in justice systems; socially dislocated)”*

(#225; PT)

*“…Yes—general developmental trajectories, underlying neurodevelopmental disorders and their interaction with persistent pain disorders, e.g., Autism …”*

(#352; OT)

Within the second area ‘Assessment and measurement of pediatric pain’ the content ‘unidimensional tools for pediatric pain intensity’ occurred most frequently (48.3%) in the curricula, and the content ‘biomarker indicators of neonatal/infant pain’ was the most frequently not covered content (35.7%). The contents with the highest rating in the category ‘very important’ were ‘assessment of pediatric pain within biopsychosocial framework’ (93.5%), and ‘impact of pediatric pain on mood, usual activities/function/quality of life/sleep/school absenteeism’ (93.5%). Further ratings with the multiple, divergent response categories are illustrated in Figure 4.

Some suggestions for further contents for this area were inclusion of the child’s environment in the assessment procedure. Furthermore, using a framework like ICF or core outcomes for assessment was recommended, e.g.,:

*“I think it’s also important to teach assessment using a conceptual framework like … interpretation of the ICF model…”*

(#133; PT)

*“…Concept of Pain Inventory (COPI) for identifying and addressing specific conceptual ‘gaps’ and misconceptions…”*

(#347; PT)

*“… too often the diagnosis is made too late and there is poor (too little) proper follow-up from the various disciplines...”*

(#248; Nursing)

In the third area ‘Management of pediatric pain’ the subheading ‘goals of pediatric pain management’ occurred most frequently (44.4%), and the content ‘massage therapy’ was the most frequently not covered content (33.3%).

The contents with the highest rating in the category ‘very important’ were ‘goals of pediatric pain management’ (88.9%) and ‘improvement of bio-, psycho-, social functioning: return to school, relationship to family, etc.’ (88.9%). Further ratings with the multiple, divergent response categories are illustrated in Figure 5.

Suggestions for the third area also placed emphasis on the inclusion of the child’s environment (parents, peers, school, sports, …) in decisions and management. Furthermore, the inclusion of psychological strategies and motivational techniques adapted to children and focusing on activation and self-management were proposed, e.g.,:

*“…Use of reward/reinforcement to increase adherence to treatment/exercise program…”*

(#222; Psychology)

*“… Yes, occupational therapists should be able to tailor activity to be both developmentally sensitive and aligned with patient interests…”*

(#352; OT)

*“...teach children to mobilize again and motivate them to move...”*

(#248; Nursing)

*“We focus on self-management strategies and do not teach or promote aquatic therapy (unless the patient is a swimmer), TENS, heat, massage or ice.”*

(#133; PT)

The two items of the area ‘Specific clinical conditions in pediatric pain populations’ were not frequently covered in the curricula: 25.9% covered ‘insight into selected clinical conditions and special child patient populations’, and 25% covered ‘pediatric pain management in different settings’. Both items were rated as ‘very important’ by 70% and 73.3%, respectively (Figure 6).

Suggestions for the fourth area ‘Specific clinical conditions in pediatric pain populations’ focused on chronic pain and on complex conditions in this vulnerable patient group, e.g.,:

*“It is important to understand the complex presentations, particularly those which are associated with high levels of misinformation, distress or stigma in the community.”*

(#352; OT)

*“Managing pain for children and adolescents with complex neurological impairment such as cerebral palsy or autism spectrum disorder.”*

(#157; OT)

*“Setting—school context—how interventions can be delivered in this setting…”*

(#222; Psychology)

The comparison of experts’ ratings and guidelines that covered this item shows large congruence. Almost all of the highest rated contents by experts (‘very important’) were also found in almost all of the consulted guidelines. Items with a low rating, e.g., “use of laboratory tests/imaging techniques” or ‘insight into the opportunities of CAM (=complement and alternative therapy interventions) for different pediatric age groups’ were also less depicted in the guidelines, see Table 3. This table compares the ratings of the survey responders with the number of guidelines that recommended this content, both shown in percentages.

## 4. Discussion

The study provides important information on pediatric pain education issues especially for PTs and OTs. To the best of our knowledge, this is one of the first studies which systematically evaluates contents for a pediatric pain education curriculum for therapists. By combining the different methods: scoping survey, consideration of guideline recommendations and the expert survey a well-founded catalogue of contents was generated.

Our first aim was to extract important contents for a pediatric pain education curriculum for specialized pain therapists from the existing literature. A broad literature search resulted in nine studies with reference to pediatric themes in PT and or OT programs, but none about a pain curriculum in sports therapy programs. Therefore, this profession was excluded from our further investigations, but should all the more be considered in further research and in practice with regard to interprofessional pediatric pain management.

Nevertheless, from the articles included in our review we could derive information about content and extent of pain education in about 278 educational pain programs in six different regions. Especially the studies from the USA and Canada [33,35,37], but also the study from Spain [40], were delivered by teams that included experts who were also task force members of the IASP interprofessional or PT curricula. We could show clearly that most of the programs include very few pediatric topics although all stated the importance of such topics for pain education. However, the results of the studies did not allow to derive in-depth information on adequate contents for a specialized pediatric pain education program.

As a consequence, and as a second step of our project, a survey among pediatric pain experts was conducted. The survey provided information about specific pediatric contents included in the curricula used or developed by the pain experts and depicted their opinion regarding the importance of such contents for a specialized curriculum. The proposed contents for the pediatric pain PT-/OT-curriculum in our questionnaire were mainly derived from recent pediatric pain guidelines, which include PT and OT recommendations. The contents were classified in line with the four domains of the IASP and EFIC curricula [21,22,23,24]. This is a common approach, also propagated by the pain curricular development experts in an IASP Factsheet regarding ‘Pain Curriculum Design Models and Implementation Approaches’ [45].

By inviting the members of three of the IASP Special Interest Groups, we gathered a representative international sample of 45 experts in pediatric pain as well as of educational experience, most of them PTs but also OTs and other professions. We had a majority of PTs compared to OTs in our sample and this is a similar phenomenon in previous curricular studies [16,40,41].

One of the key findings from the survey is that actually there is insufficient specific education for OTs and PTs on the management of pediatric pain. There were hardly any curricula for pediatric pain present and if pediatric pain is covered, then this was limited to a few hours. This is in line with the results of our review, where several studies also reported a low coverage of pediatric themes and inadequate time spent on this topic [33,36,37,38,40]. We also found that little of the contents were included in the curricula mentioned by the experts, if available. It seems that a basic training on general pain management is often prioritized. But given the growing prevalence of pediatric pain at the very least an introduction to this problem should be part of the curricula for standard training to raise awareness among the students for this issue. It is to be expected that only a small number of students are interested to go into depth of this topic, so it seems sensible to build on these introductory hours in subsequent stand-alone specialization courses. The provision of postgraduate specialization courses and eventually online specialized pain education resources could be a solution to cover the deficits on pediatric pain management among OTs and PTs, but there are few specialization courses at present [14]. One interesting online pediatric pain curriculum based on the interprofessional IASP Curriculum [21] is currently available at the homepage of SickKids [46]. Another learning curriculum offers free accessible, downloadable guidance [47]. Both addressed not specific PTs/OTs but all health care providers who are interested in pediatric pain management.

With regard to the survey results we were able to draw on expertise and on evidence-based clinical reasoning of pediatric pain. In each of the four areas, the most important contents were clearly highlighted and the highest rated contents were also the contents that were represented in all the guidelines.

Contents regarding active interventions (e.g., graded exposure) were considered more important in comparison to passive interventions (e.g., massage), which is in line with best evidence recommendations for pediatric pain rehabilitation [11]. Exercise therapy guided by PTs and/or OTs has become an integral part of interdisciplinary pediatric pain programs [48]. In fact, there is little evidence for passive techniques, yet some specific pediatric populations may benefit from them. Massage therapy may reduce pain and anxiety among children with cancer [49,50]. There is also growing evidence regarding skin-to-skin contact especially to reduce procedural pain in neonates and infants [51,52,53,54].

It was encouraging to see that the biopsychosocial approach is of central relevance for almost all responders. It is state-of-the-art to assess biological, psychological, and sociocultural information to plan an adequate multimodal pediatric management [47]. Even when a physical component such as generalized joint hypermobility has been demonstrated in adolescents, psychosocial factors (e.g., catastrophizing thoughts, pain-related anxiety) appear to have an impact on the development or maintenance of chronic musculoskeletal pain [55]. Furthermore, educational curricula addressing psychosocial aspects and considering the children’s life situation, seem to better prepare therapists for their health-focused practice [56].

Particularly important contents for the pediatric target group were clearly identified, e.g., to assess the “impact of pediatric pain on mood, daily activities, school absenteeism, quality of life”. Or in the area “pain management” some pediatric specific goals were considered very important, e.g., improvement of functioning: return to school, relationship to family and peers, etc. These are all recommended contents in pediatric pain rehabilitation [57,58].

Especially the answers to the open questions were able to map that the responding experts put a special emphasis on the differences in pain presentation and handling with a pediatric population compared to adults. Pediatric patients should not be treated like small adults, they need a specialized assessment and management [54,58], and an adequate interaction and communication between therapists-child -parents is imperative [59,60].

Children/youth adapted communication style and the use of informative adapted materials suitable for children and parents were often recommended among the experts in our survey, what is also reflected in the current literature [59,60]. For an adapted education about pain neuroscience vivid, child-friendly materials are available in different languages, e.g., an educational video ”Understanding pain—and what’s to be done about it in 10 min!” [61], a cartoon book ”A Journey to Learn about Pain” [62,63,64], or a pain neuroscience education program for children “PNE4Kids” [65,66].

When interpreting the results, it should be noted that this study is not exempt from limitations. First, as the survey was distributed anonymous via the IASP special interest groups and individual private networks, we could not calculate an exact response rate, because it is not known how many from those invited responded to the questionnaire. Further, it should be considered that data based on self-reporting can give a risk of bias; however, the survey was completely anonymous. The quality of an expert panel’s judgement is highly dependent on the composition of the sample [18]. Based on characteristics and quality of open comments on the questionnaire of this current expert sample, a high level of expertise on pediatric pain education and pediatric pain management could be assumed.

Second, it was not compulsory to answer all the questions, the respondents (*n* = 45) could skip some questions of the survey. This resulted in varying numbers of responders on the questions regarding covering of items in the curricula or importance of our proposed contents. Although our results focused mainly on chronic pediatric pain, the prevention of pain and timely interventions for acute pain in children should certainly not be ignored. Pain experiences in childhood can make children vulnerable to chronic pain in adulthood [5]. Non-pharmacological interventions are effective in the prevention of pediatric pain and exercise/physical activity (PT and OT) find their rightful place in the treatment of acute pain [67,68].

Furthermore, we could not derive the ideal amount of hours needed for a specialization PT-/OT-program for pediatric pain, as the literature depicted very heterogeneous numbers. The number of hours indicated by the experts between 7 and 12 h seem low for such a complex subject. Based on many years of experience in the teaching of pain management, the authors would recommend a minimum of 20 to 30 h, provided the participant PTs/OTs have previously completed a general basic pain course of at least 80 h.

But overall, both results, those from the review and those from the survey, illustrate a severe underrepresentation of pediatric pain topics in the existing curricula. Fortunately, the results of the survey depicted also clearly that the respondents highly agree to the necessity for a pediatric pain education curriculum for PTs and OTs. Specialized training is essential for improving pediatric pain management programs. In the meantime the integration of pain specialized PTs and OTs in the interdisciplinary multimodal pain teams is demanded to meet the quality standards for adults’ patients pain programs [69]. Also, in the pediatric pain teams the expertise of PT s and OTs becomes more and more relevant [70].

Regarding future lines it should be considered that this study involves only a first step in the direction of a specialized curriculum development. The evaluated contents could be integrated in a curricular model. Currently different models and perspectives for curricular development exist, e.g., a theoretical framework especially for health care providers education as proposed in the publication of Steketee et al. [71]. They described a curriculum with 4 dimensions with the following recommendations: identify the practice needs, define the capabilities, consider the teaching modus and the institutional structure.

## 5. Conclusions

This study was an important project to evaluate adequate contents for a specialized pediatric pain curriculum for PTs and OTs. The three components (review of the literature; guideline recommendations; experts’ survey) of this study provide a consistent picture that indicates an urgent need for action. The review and the survey reveal the almost non-existent opportunities for pediatric pain specialization for PTs and OTs and, if present in the curricula, then only with very small hourly amounts. The recommendations from the guidelines and those of the experts in the survey provide homogenous, concrete recommendations for required contents, which now need to be systematically transferred into a curriculum.

Furthermore, the project reflects a clear recognition about the specialized needs in the management of pediatric patients and their environment. Especially in the survey, the experts emphasized the importance of involving the parents, school teachers, and peers in the pain management decisions. Further competencies related to communication and educational skills in handling with these vulnerable patients were recommended.

It would be highly recommended to integrate general pain management themes with a short introduction about pediatric pain already in the undergrade educational level for PTs and OTs. An in-depth specialized pediatric pain education should be delivered as a stand-alone course (for experienced professionals), requiring basic knowledge and skills about pain in general. Our survey shows a clear need to build up such a specific curriculum according to the four IASP domains, focused to and enriched by specific pediatric pain issues, e.g., (1) multidimensionality of pediatric pain; (2) specific assessment of pediatric pain; (3) management of pediatric pain (4) clinical conditions in pediatric pain populations. Considering there may not be a very large population of PTs and OTs interested in a pediatric pain specialization course, an online course could also be taken into consideration. Further research and curriculum work is highly recommended, and interdisciplinary as well as international networking could lead to efficient use of knowledge, experiences, and resources.

## Figures and Tables

**Table 1:** Characteristics and main findings of included studies.

Author	Aim	Country	Education Level	Accreditation	Evaluation years	Reference to IASP Pain Curricula	Discipline	Duration	Reference to Pediatric Pain Contents	Focus	Deficits Described	Survey/Interview Description & Poss. Response Rate
Hoeger Bement & Sluka, 2015 [33]	To determine the extent of pain education in current Doctorate of PTschools.	USA	Doctoral level	Yes	2012–2013	IASP PTcurr. & 4 IASP Domains.	PT	Contact hours: mean 31 h ± 1.8 (SD) range: 5–115 h stand-alone course: *n* = 11 (6%).	Lowest coverage: assessment of pain across the life span (68%).	Basic science mechanisms/pain concepts/pain assessment & management/adequacy of pain curriculum.	Pain management in the young/46% were aware of the IASP curriculum.	Survey consisted of 10 questions/addressing directors or appropriate persons; *n =* 216 PT schools; response = 62% (*n* = 137).
Rochman et al. 2013 [34]	To test OT students’ pain knowledge and to determine specific topics to improve existing OT curricula.	USA	Master’s-level	---	2004–2009	IASP OT/PT & inter-professional curr.	OT	2 sessions of 2-h pain course embedded in a 13 weeks procedural clinical reasoning class & additional examples of clinical pain management in the course.	Correct answers: regarding children’s pain sensitivity & pain memory: 59.8% in pre- & 80.9% in post-test.	- Basics of pain management: misconceptions, myths, theories, taxonomy definitions, measurements, - OT & pain management: assessment methods/OTs role in pain team/case study.	Pain in special populations, e.g., children/ there is need for lecturers with special expertise in pain.	Survey of pain knowledge and attitudes prior to and following a pain course using the COBS adapted for OT & PT; *n* = 194 first-year OT students of *n* = 1 university response = 100%.
Hoeger Bement et al. 2014 [35]	To identify how core competencies for pain can be applied to the PT curriculum.	USA & Canada	Entry level	---	---	IASP PT & inter-professional curr. & 4 IASP Domains.	PT	Integration of competencies into a PT curriculum & additional stand-alone course on pain recommended.	Using specific pain assessments for special patient populations e.g., infants & improving pain management throughout the life span.	Description of PT core competencies for prelicensure education in pain management.	Innovative evidence based initiatives.	Based on expert consensus-derived pain management core competencies for health professional education.
Scudds et al. 2001 [36]	To determine the current status of pain topics in PT curricula.	North America & Canada	Master’s-level	Yes	1997	IASP OT & PT curr.	PT	Modal amount of time spent on pain = 4 h & 11/107 stand-alone courses.	23.8% stated adequate time spent on pain in children.	Basic science mechanisms/pain assessment & management/time spent on pain topics.	Knowledge of curr. (21.9%); inadequate time for pain topics (43.3%) & pediatric pain inadequately covered (76.2%).	Survey of *n* = 169 PT programs using the Pain Survey Instrument/ target population = program directors or faculty members/ response rate = 63.3% (*n* = 107/169).
Hunter et al. 2008 [37]	To describe innovations based on comprehensive evaluations of outcomes of IPC implementation.	Canada (Toronto)	Undergraduate level	---	2002–2007	IASP core & discipline-specific curr.	OT/PT & other disciplines.	Mandatory, stand-alone course = 20 h & 1 uni-professional discipline-specific session.	Optional: pain in children (1 h30) 1 case focus on childhood arthritic pain.	A flexible curriculum: choice of sessions/variety of patient cases/reduced didactic hours.	Students applied their learning in a surrogate clinical situation.	Survey among students, clinician-facilitators and faculty. Including the Daily Content and Process Questionnaire/Pain Knowledge & Beliefs Questionnaire & Comprehensive Plan Evaluation/*n =* 6 Health Science Faculties.
Wideman et al. 2020 [38]	To determine the current state of pain education across PT programs.	Canada	Master’s-level	Yes	2016	IASP PT curr.; 3 IASP Domains.	PT	Median 18 h (range 8–65 h) stand-alone course in 2 programs.	28.6% addressed assessment & management of pediatric pain in sufficient depth.	% of items addressed in the programs: Domain1= 35.7%; Domain 2 = 50%; Domain 3 = 35.7%	Integration of curriculum themes: 38.6% full; 52.6% partial; 10.5% not.	Survey of all the national PT programs (=14), targeting 57 themes *n =* 16 pain educators.
Hush et al. 2018 [39]	To describe the embedding of the 2012 IASP PT curriculum into a PT program.	Australia	Doctoral level	---	2012–2017	IASP PT curr. (2012) & 4 IASP Domains.	PT	Integrated in the PT curriculum.	Skills training emphasises the provision of … evidence-based health care across the lifespan.	95 items of IASP curriculum successfully embedded/Special tools: six core concepts of pain & a clinical model of pain education.	Provision of training and resources to upskill teachers is critical.	Special tools developed with leading pain education specialists. Evaluation: students’ knowledge & skills: NPQ-R, APP tool, Mastery Checklist & clinical simulation exams; *n =* 1 PT program.
Miro et al. 2019 [40]	To describe the content of pain curricula in healthcare & veterinary education programs.	Spain: Catalonia	Undergraduate level	Yes	2018	IASP inter-professional curr. & 4 IASP Domains.	OT/PT & other disciplines.	84% courses mandatory PT: M = 97 h (SD = 34; range = 34–129); OT: M= 13 h (SD = 9; range 7–20).	4% embedded pediatric pain PT: *n* = 2 (2%) OT: *n* = 1 (6.7%) (*n =* number of responders).	Overview pain contents in 10 disciplines IASP curr contents: Domain 1= 17%; Domain 2= 21%; Domain 3= 10%; Domain 4= 14%.	Pain education not adequate & no interprofessional program.	Survey included 31 questions/addressing *n* = 1564 course leaders of *n =* 11 universities response *n =* 550 (35%).
van Lankveld et al. 2020 [41]	To describe needed competency levels for an interprofessional pain education core curriculum.	NL national & international panels	Undergraduate level	---	2016	IASP inter-professional pain curr. (2012) 4 IASP Domains.	OT/PT & other disciplines.	Targeted is a stand-alone interprofessional pain education course.	Pediatric pain was excluded due to <70% agreement.	% of items selected for: Domain 1 = 66.6%; Domain 2 = 78%; Domain 3= 76%; Domain 4= 41%.	Exclusion of pain in infants although 79% of panel b rated inclusion.	7 national (panel 1) & 15 international experts (panel 2) rated the curriculum items; inclusion of items with >70% agreement.

APP Tool = Assessment of Physiotherapy Practice tool; COBS = City of Boston’s Rehabilitation Professionals’ Knowledge and Attitude Survey Regarding Pain; curr = curriculum/curricula; IASP Domains: 1 = multidimensional nature of pain, 2 = pain assessment and measurement, 3 = management of pain, 4 = clinical conditions; IPC = Interfaculty Pain Curriculum; M= mean NPQ-R: Neurophysiology of Pain Questionnaire (revised); OT = occupational Therapy; PT = Physiotherapy; OT = Occupational Therapy; --- = not specified.

**Table 2:** Critical Appraisal of the studies included in the scoping review.

Authors	Hoeger Bement & Sluka, 2015	Rochman et al. 2013	Hoeger Bement et al. 2014	Scudds et al. 2001	Hunter et al. 2008	Wideman et al. 2020	Hush et al. 2018	Miro et al. 2019	van Lankveld et al. 2020
Reference	[33]	[34]	[35]	[36]	[37]	[38]	[39]	[40]	[41]
Introduction									
1. Were the aims/objectives of the study clear?	√	√	√	√	√	√	√	√	√
Methods									
2. Was the questionnaire clearly described or is the questionnaire available?	√	√	n/a	√	√	√	n/a	√	√ *
3. Was the way of contacting the respondents described?	√	√	n/a	√	√	√	n/a	√	√
4. Was a pilot test conducted?	√	n/a	n/a	√	n/a	√	n/a	√	*
5. Was there a description of the data analysis?	√	√	n/a	p	√	√	n/a	√	√
Results									
6. Was there a description of the characteristics of the survey responders?	√	p	n/a	p	√	p	n/a	√	√
7.Was the response rate depicted?	√	√	n/a	√	√	√	n/a	√	√
8. Were the results presented clearly and comprehensibly?	√	√	√	√	√	√	√	√	√
Discussion									
9. Were the authors’ discussions and conclusions justified by the results?	√	√	√	√	√	√	√	√	√
10. Were the limitations of the study discussed?	√	p	n/a	√	p	√	n/a	√	√

n/a= not applicable; p = partially; [34] & [35] n/a= due to the use of a validated standardized questionnaires; [37] n/a= no survey; only reflections about the implementation of pain management core competencies into the PT-curriculum; [39] n/a= no survey; description of the embedding of the IASP pain curriculum in a PT program; [41] * = a modified Delphi method was used.

**Table 3:** Comparison of experts’ ratings and guidelines.

**1. Multidimensional Nature of Pediatric Pain**	**Ratings (%)**	**Guidelines (%)**
Epidemiology of pediatric pain	53.1	100
Theories of pediatric pain		
● Biopsychosocial model & pediatric pain	87.5 *	100 *
● Definition of pediatric pain	71.9	85.7
● Classification systems of pediatric pain	53.1	71.4
Pain mechanism in pediatric pain		
● The multidimensional nature of pediatric pain	87.5 *	100 *
● Implications of pediatric pain	78,1	71.4
● Consequences of unrelieved pediatric pain	78.1	48.9
Ethical principles & pediatric pain		
● Ethical standards of care for pediatric pain	71.9	57.1
● Inadequate pain management for the different age groups: premature babies, neonates, infants, children & adolescents	84.4 *	85.7 *
**2. Assessment and measurement of pediatric pain**		
Assessment of pediatric pain within the biopsychosocial framework	93.5 *	100 *
Interprofessional collaboration	87.1	100
● Comprehensive assessment of complex pediatric pain problems	90.3 *	100 *
● Consideration of appropriate assessment and measurement methods for neonates, infants, children and adolescents including parents if necessary	83.9	100
History taking of pediatric pain		
● Pediatric pain location, grade of pain, contributing factors, etc.	77.4	83.3
● Impact of pediatric pain on mood/daily activities/school absenteeism/quality of life	93.5 *	100 *
● History of previous pediatric pain and treatment	80.6	83.3
● Possible comorbidities influencing pediatric pain	83.9	83.3
● Characteristics of the child/adolescent/familian occurrence of symptoms, environment	80.6	100
Physical examination of pediatric pain	71.0	66.7
Investigations: Laboratory test/Imaging techniques etc.	**35.5** **^§^**	**50.0** **^§^**
Use of child adapted standardized ools/questionnaires/assessment instruments	70.9	100
Use of parents adapted tools/questionnaires/assessment instruments	77.4	66.7
**3. Management of pediatric pain**		
Goals of pediatric pain management:	88.9 *	100 *
● Prevention and/or reduction of pediatric pain intensity	57.9	100
● Improvement of bio-, psycho-, social functioning (return to school, relationship to family etc.)	88.9 *	85.7 *
Pediatric pain management planning decisions	73.7	100
● Develop and monitor a child- (& parents-) centred management plan including realistic goals	72.2	100
● Provide child (& parents) adapted information/education	84.2 *	100 *
Treatment considerations adapted to the different pediatric age groups	80.0	100
● Specialized pain education for children (including communication & motivational techniques	84.2 *	100 *
● Physical interventions adapted for different pediatric age groups	73.7	100
● Exercise interventions for pediatric pain, e.g., Graded exposure	84.2 *	100 *
● Insight into the opportunities of psychological interventions adapted to different pediatric age groups	65.0	100
● Insight into the opportunities of medical/pharmacological interventions adapted to different ages	59.1	100
● Insight into the opportunities of CAM (=complement & alternative therapy interventions) for different pediatric age groups	**50.0** **^§^**	**42.9** **^§^**
**4. Specific clinical conditions in pediatric pain populations**		
Insight into selected clinical conditions and special child patient populations	70.0	85.7
Pediatric pain management in different settings: e.g., primary care, secondary care, pediatric pain clinics, etc.	73.3	85.7

* = highest quotes; ^§^ = lowest quotes.

## Data Availability

Materials of the expert survey are available from the corresponding author on reasonable request.
